# Machine learning for identifying benign and malignant of thyroid tumors: A retrospective study of 2,423 patients

**DOI:** 10.3389/fpubh.2022.960740

**Published:** 2022-09-14

**Authors:** Yuan-yuan Guo, Zhi-jie Li, Chao Du, Jun Gong, Pu Liao, Jia-xing Zhang, Cong Shao

**Affiliations:** ^1^Department of Laboratory Medicine, Chongqing General Hospital, Chongqing, China; ^2^Department of Laboratory Medicine, Fuling Center Hospital of Chongqing City, Chongqing, China; ^3^Department of Information Center, University-Town Hospital of Chongqing Medical University, Chongqing, China; ^4^Department of Breast and Thyroid Surgery, Chongqing General Hospital, Chongqing, China

**Keywords:** thyroid tumor, machine learning, predictive model, *BRAFV600E* gene mutation, risk-factors

## Abstract

Thyroid tumors, one of the common tumors in the endocrine system, while the discrimination between benign and malignant thyroid tumors remains insufficient. The aim of this study is to construct a diagnostic model of benign and malignant thyroid tumors, in order to provide an emerging auxiliary diagnostic method for patients with thyroid tumors. The patients were selected from the Chongqing General Hospital (Chongqing, China) from July 2020 to September 2021. And peripheral blood, *BRAFV600E* gene, and demographic indicators were selected, including sex, age, *BRAFV600E* gene, lymphocyte count (Lymph#), neutrophil count (Neu#), neutrophil/lymphocyte ratio (NLR), platelet/lymphocyte ratio (PLR), red blood cell distribution width (RDW), platelets count (PLT), red blood cell distribution width—coefficient of variation (RDW–CV), alkaline phosphatase (ALP), and parathyroid hormone (PTH). First, feature selection was executed by univariate analysis combined with least absolute shrinkage and selection operator (LASSO) analysis. Afterward, we used machine learning algorithms to establish three types of models. The first model contains all predictors, the second model contains indicators after feature selection, and the third model contains patient peripheral blood indicators. The four machine learning algorithms include extreme gradient boosting (XGBoost), random forest (RF), light gradient boosting machine (LightGBM), and adaptive boosting (AdaBoost) which were used to build predictive models. A grid search algorithm was used to find the optimal parameters of the machine learning algorithms. A series of indicators, such as the area under the curve (AUC), were intended to determine the model performance. A total of 2,042 patients met the criteria and were enrolled in this study, and 12 variables were included. Sex, age, Lymph#, PLR, RDW, and BRAFV600E were identified as statistically significant indicators by univariate and LASSO analysis. Among the model we constructed, RF, XGBoost, LightGBM and AdaBoost with the AUC of 0.874 (95% CI, 0.841–0.906), 0.868 (95% CI, 0.834–0.901), 0.861 (95% CI, 0.826–0.895), and 0.837 (95% CI, 0.802–0.873) in the first model. With the AUC of 0.853 (95% CI, 0.818–0.888), 0.853 (95% CI, 0.818–0.889), 0.837 (95% CI, 0.800–0.873), and 0.832 (95% CI, 0.797–0.867) in the second model. With the AUC of 0.698 (95% CI, 0.651–0.745), 0.688 (95% CI, 0.639–0.736), 0.693 (95% CI, 0.645–0.741), and 0.666 (95% CI, 0.618–0.714) in the third model. Compared with the existing models, our study proposes a model incorporating novel biomarkers which could be a powerful and promising tool for predicting benign and malignant thyroid tumors.

## Introduction

The incidence of thyroid tumors has been increasing over the past 20 years, and it was the eighth most commonly diagnosed tumors in the world among endocrine tumors (1–3). According to the National Cancer Registry, thyroid tumors in China will continue to grow at an annual rate of 20% (4, 5). Therefore, identifying benign and malignant tumors owns great significance for early clinical intervention and treatment. Although ultrasonography and fine needle aspiration biopsy (FNAB) cytology methods can diagnose most thyroid tumors, there were still some patients who were misdiagnosed or overtreated. In addition, the limitations of those examinations included the need for a highly experienced cytopathologist for accurate interpretation, and not suitable for early screening of disease.

At present, many biomarkers of thyroid tumors have been discovered by researchers. Ozmen found that higher NLR and PLR were associated with worse survival in differential thyroid tumors (6). Another study from Turkey suggested that mean platelet volume (MPV) levels can be used as an easily available biomarker for monitoring the risk of papillary thyroid carcinoma (PTC) in patients with thyroid nodules, enabling early diagnosis of PTC (7). And Liu found that lower pretreatment platelet count (PLT) levels may indicate a poor prognosis for PTC (8). In particular, the *BRAFV600E* gene is also an important biomarker for the occurrence and progression of papillary thyroid tumors (9). In addition, the review by Qian and Iryani mentions that many genetic biomarkers can differentiate benign from malignant thyroid tumors (10, 11). However, most studies just investigated the diagnostic performance of individual biomarkers, and few studies have integrated these biomarkers to construct models that can be used to diagnose benign and malignant thyroid tumors in clinical practice. Previous studies have the shortcomings of small sample size and large differences in diagnostic performance between different biomarkers.

Machine learning (ML) is an emerging artificial intelligence discipline that analyzes multiple data types and uses them to explore disease risk factors, accurate diagnosis, and prognosis (12). Moreover, it can integrate multiple clinical indicators, explore the nonlinear relationship between predictors and clinical outcomes, and solve problems such as poor performance of logistic methods in traditional clinical modeling. Sui developed a deep-learning AI model (ThyNet) using ultrasound images to differentiate between malignant tumors and benign thyroid nodules with an AUC of 0.875 (95% CI, 0.871–0.880) (13). Although there have been some studies using ML algorithms to diagnose benign and malignant thyroid tumors, the data selected are mostly image data, which makes data collection more complicated.

Therefore, this study aims to apply ML algorithms to build a predictive model of thyroid tumors with demographic, peripheral blood laboratory, and genetic biomarkers to provide an accurate and reliable prediction method for the early discrimination of benign and malignant thyroid tumors.

## Methods

### Study participants

Patients with thyroid tumor included in the current study, were selected from the Chongqing General Hospital (Chongqing, China) from July 2020 to September 2021. According to WHO 2017 classification and the eighth edition of the AJCC/TNM classification (TNM-8) (14), operating records and final pathologic reports were reviewed to ascertain tumor categories, they were divided into benign groups and malignant groups. Benign groups are defined as thyroid follicular nodular disease, follicular adenoma, follicular adenoma with papillary architecture, oncocytic adenoma of the thyroid, and benign thyroid nodules. While, malignant groups are defined as follicular thyroid carcinoma, invasive encapsulated follicular variant papillary carcinoma, papillary thyroid carcinoma, oncocytic carcinoma of the thyroid, follicular-derived carcinomas, high-grade, and anaplastic follicular cell-derived thyroid carcinoma (15).

This study was exempt from ethical review by the Institutional Review of the Chongqing General Hospital. The study methods were carried out in accordance with the relevant guidelines and regulations.

### Candidate predictors

The data was collected from the electronic medical record (EMR) system of the Chongqing General Hospital, which contains laboratory examination records, diagnosis and treatment process records, doctor orders, etc. Patient's peripheral blood indicators, *BRAFV600E* gene, and demographic indicators were selected, including age, sex, lymphocyte count (Lymph#), neutrophil count (Neu#), red blood cell distribution width (RDW), red blood cell distribution width - coefficient of variation (RDW–CV), platelets count (PLT), neutrophil/lymphocyte ratio (NLR), platelet/lymphocyte ratio (PLR), alkaline phosphatase (ALP), parathyroid hormone (PTH), and *BRAFV600E* gene mutation as predictors to build a ML model to identify benign and malignant thyroid tumors. All the peripheral blood tests and *BRAFV600E* gene results were obtained at the first examination after the patient was admitted to the hospital.

The *BRAFV600E* gene mutation was detected by real-time PCR using the ABI QuantStudio^®^5 Real-Time PCR System, according to the manufacturer's instructions (Human BRAFV600E Mutation assay Kit, YZY MED, Wuhan, China) The DNA from FNAB specimen was extracted using a companion kit, which was provided by the same manufacturer. The DNA concentration was quantified in a Nano-300 Micro Spectrophotometer (ALLSHENG Instrument Co., Ltd. Hangzhou, China) as per the manufacturer's instructions. The DNA was immediately used to carry out the test of *BRAFV600E* gene mutation.

### Statistical analysis

All the statistical analyses and model building were conducted in R for windows (version 4.0.1, https://www.r-project.org/). For information on hardware devices in the development environment, please see Supplementary Table 1.

The data were presented as count with percentage for categorical variables, median with interquartile range (IQR), or mean with SD for continuous variables. For the variables with miss rate <30%, missforest algorithm was used to fill. First, the Mann–Whitney *U*-test or *t*-test was performed for the continuous variables, and the chi-square test for categorical variables was carried out used for univariate analysis. The variables after univariate analysis were analyzed by the least absolute shrinkage and selection operator (LASSO). Afterward, random forest (RF), extreme gradient boosting (XGBoost), light gradient boosting machine (LightGBM) and adaptive boosting (AdaBoost) were used to establish prediction models. We used the grid search algorithm to find the optimal parameters of each algorithm to optimize the performance of the model. Sensitivity (SEN), specificity (SPE), precision, recall, F1, and the area under the curve (AUC) were intended to determine the model performance.

## Result

### Sample collection

**Figure 1:**
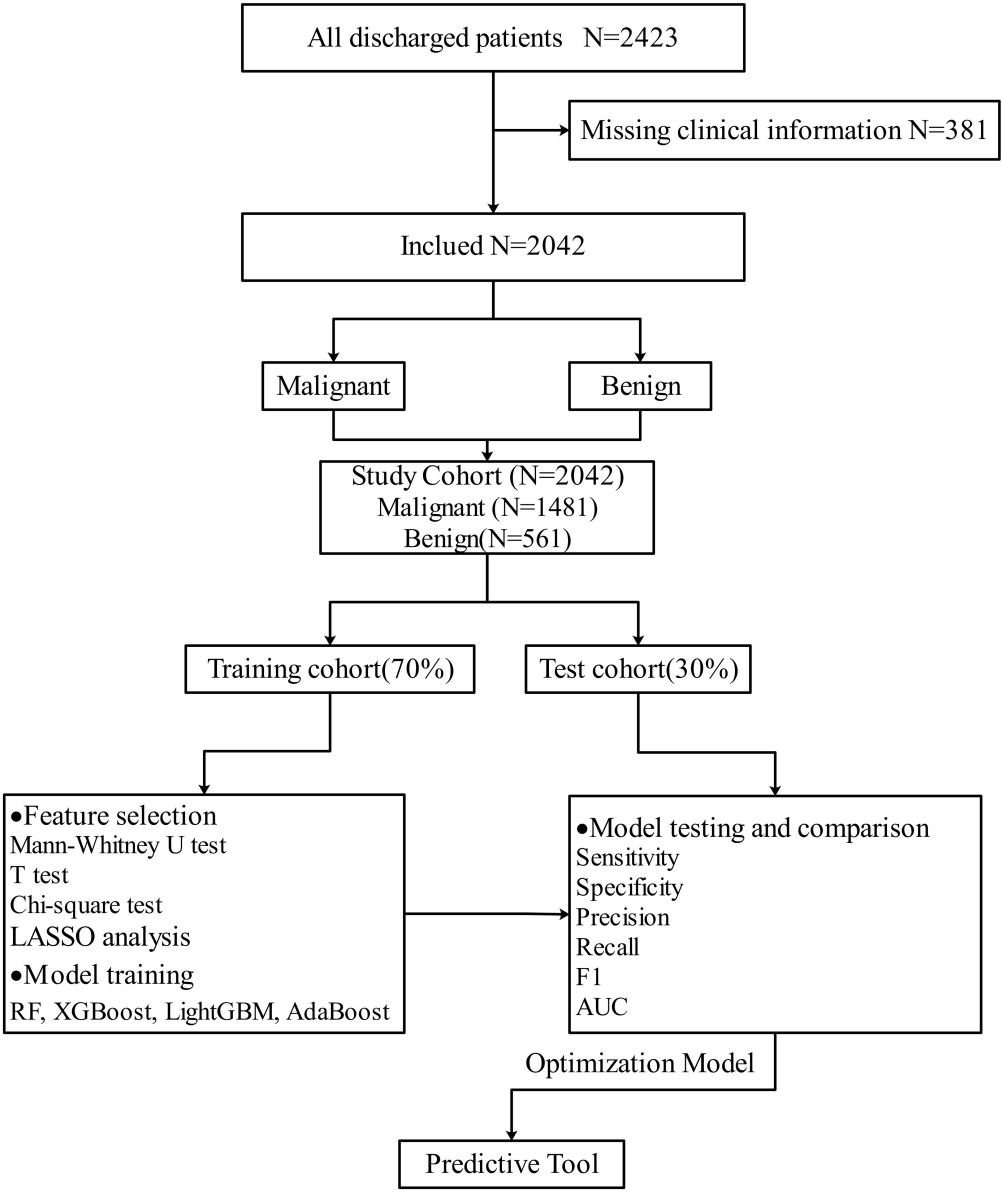
Flowchart of research object.

A total of 2,423 patients met the inclusion criteria and were enrolled in the study. In total, 381 patients were excluded due to missing clinical data. At last, a total of 2,042 patients with 12 predictors were included in the final study. Table 1 shows the information of the whole cohort. In the whole cohort, 1,481 malignant patients and 561 benign patients were included. The average age of patients was 42.03 ± 11.30 years, ranging from 14 to 76 years, women accounted for 77.34% (1,580 cases) and men 22.66% (463 cases). The specific screening process and study protocol are shown in Figure 1.

**Table 1:** Clinical characteristics and variables of patients in all cohorts.

**Predictors**	**Benign** **(*N* = 561)**	**Malignant** **(*N* = 1,481)**	***P*** **-value**
**Sex (%)**			
Male	105 (18.7)	357 (24.1)	0.011
Female	456 (81.3)	1,124 (75.9)	
**BRAFV600E (%)**			
Mutation	76 (13.5)	1,170 (79.0)	<0.001
Wild	485 (86.5)	311 (21.0)	
Age (years)	45.00 [35.00, 52.00]	39.00 [32.00, 50.00]	<0.001
Lymph# ( ×10^9^/L)	1.64 [1.37, 2.01]	1.58 [1.29, 1.94]	<0.001
Neu# ( ×10^9^/L)	3.64 [2.85, 4.65]	3.60 [2.84, 4.57]	0.991
NLR	2.13 [1.69, 2.85]	2.20 [1.70, 2.96]	0.061
PLR	130.06 [103.38, 157.24]	140.00 [110.36, 172.27]	<0.001
RDW (%)	42.30 [40.60, 43.90]	41.90 [40.50, 43.40]	0.002
PLT ( ×10^9^/L)	215.00 [184.00, 251.00]	222.00 [187.00, 260.00]	0.061
RDW-CV	12.90 [12.50, 13.40]	12.80 [12.50, 13.30]	0.594
ALP (U/L)	67.00 [59.00, 78.14]	67.00 [56.00, 81.00]	0.395
PTH (ng/ml)	49.20 [43.90, 53.75]	48.50 [37.80, 58.90]	0.786

### Model building

**Figure 2:**
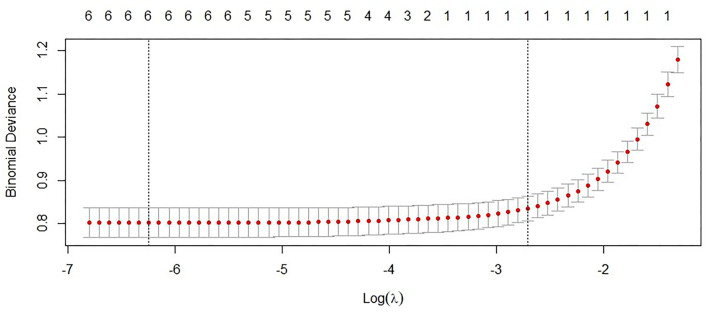
LASSO analysis of indicators after univariate analysis.

The data were split into a training cohort (70%, *N* = 1,429) and a test cohort (30%, *N* = 613) by random number table. In the training cohort, there were 395 cases of the benign group and 1,034 cases of the malignant group. In the test cohort, there were 166 cases of the benign group and 447 cases of the malignant group. The predictors we collected were used as input variables of ML algorithms. Whether malignancy or benign was regarded as the outcome event (yes = 1, no = 0) to establish prediction model by using training cohort, and the test cohort was used to verify the ability of the established prediction model previously. According to Table 2, univariate analysis results indicated that 6 predictors were statistically significant between the malignant group and benign group in training cohort. We performed the LASSO analysis on the 6 indicators with statistically significant, and the results showed that these 6 indicators were all selected by LASSO (Figure 2). Therefore, our final diagnostic model included the 6 indicators of sex, age, Lymph#, PLR, RDW, and BRAFV600E.

**Table 2:** Clinical characteristics and variables of patients in training cohort and test cohort.

**Predictors**	**Training cohort**			**Test cohort**		
	**Benign (*N* = 395)**	**Malignant (*N* = 1,034)**	***P*** **-value**	**Benign (*N* = 166)**	**Malignant (*N* = 447)**	***P*** **-value**
**Sex (%)**						
Male	70 (17.7)	247 (23.9)	0.015	35 (21.1)	110 (24.6)	0.421
Female	325 (82.3)	787 (76.1)		131 (78.9)	337 (75.4)	
**BRAFV600E (%)**						
Mutation	55 (13.9)	822 (79.5)	<0.001	21 (12.7)	348 (77.9)	<0.001
Wild	340 (86.1)	212 (20.5)		145 (87.3)	99 (22.1)	
Age (years)	45.00 [36.00, 52.00]	39.00 [33.00, 50.00]	<0.001	44.00 [34.00, 52.00]	38.00 [32.00, 49.00]	0.008
Lymph# ( ×10^9^/L)	1.64 [1.39, 2.00]	1.56 [1.28, 1.92]	0.001	1.65 [1.35, 2.05]	1.61 [1.31, 1.96]	0.189
Neu# ( ×10^9^/L)	3.62 [2.83, 4.65]	3.58 [2.83, 4.54]	0.925	3.66 [2.93, 4.66]	3.64 [2.88, 4.63]	0.877
NLR	2.14 [1.69, 2.91]	2.21 [1.71, 2.98]	0.074	2.11 [1.70, 2.73]	2.18 [1.70, 2.95]	0.48
PLR	131.40 [103.99, 160.30]	140.70 [110.93, 173.62]	<0.001	127.47 [101.35, 155.48]	138.33 [109.73, 170.43]	0.013
RDW (%)	42.40 [40.90, 44.00]	41.90 [40.40, 43.48]	<0.001	41.95 [40.30, 43.58]	41.90 [40.50, 43.20]	0.816
PLT ( ×10^9^/L)	215.00 [185.00, 253.00]	221.00 [186.00, 259.00]	0.222	215.00 [183.00, 248.75]	225.00 [190.00, 261.00]	0.121
RDW-CV	12.90 [12.50, 13.40]	12.80 [12.50, 13.30]	0.387	12.80 [12.40, 13.20]	12.80 [12.50, 13.30]	0.709
ALP (U/L)	67.00 [59.26, 78.28]	66.80 [56.00, 80.89]	0.23	66.44 [58.77, 78.00]	68.00 [56.00, 82.00]	0.791
PTH (ng/ml)	49.03 [43.50, 53.73]	48.70 [37.80, 58.80]	0.925	49.44 [44.19, 53.82]	47.68 [37.90, 59.45]	0.498

We built 3 ML models with different predictors, the first model included all the predictors we included, the second model included predictors after feature selection, and the third model included patient peripheral blood predictors. For the specific construction steps of the model, please see Supplementary Figure 1, and the detailed description of the three models can be found in Supplementary Table 2. In addition, we also used the grid search algorithm to find the optimal parameters of the ML algorithm. The grid search algorithm permutes and combines each possible parameter value, and then substitutes the results of all possible combinations into the algorithm for model training. The optimal parameter combination was selected from all possible parameter combinations. In our research, we selected the optimal parameters of four ML algorithms: RF, XGBoost, LightGBM, and Adaboost through the grid search algorithm. Please see Table 3 for the optimal parameters of each algorithm.

**Table 3:** The optimal parameters of the three models.

**Categories**	**Algorithm**	**Parameter**
The first model	RF	mtry = 1, ntree = 60, nodesize = 8
	XGBoost	max_depth = 3, eta = 0.6, nrounds = 5
	LightGBM	nrounds = 20, min_data = 1, learning_rate = 0.1
	AdaBoost	mfinal = 170
The second model	RF	mtry = 6, ntree = 140, nodesize = 12
	XGBoost	max_depth = 4, eta = 0.3, nrounds = 3
	LightGBM	nrounds = 10, min_data = 3, learning_rate = 0.1
	AdaBoost	mfinal = 20
The third model	RF	mtry = 1, ntree = 90, nodesize = 10
	XGBoost	max_depth = 6, eta = 0.7, nrounds = 3
	LightGBM	nrounds = 10, min_data = 3, learning_rate = 0.4
	AdaBoost	mfinal = 5

### Performance evaluated in different models

**Figure 3:**
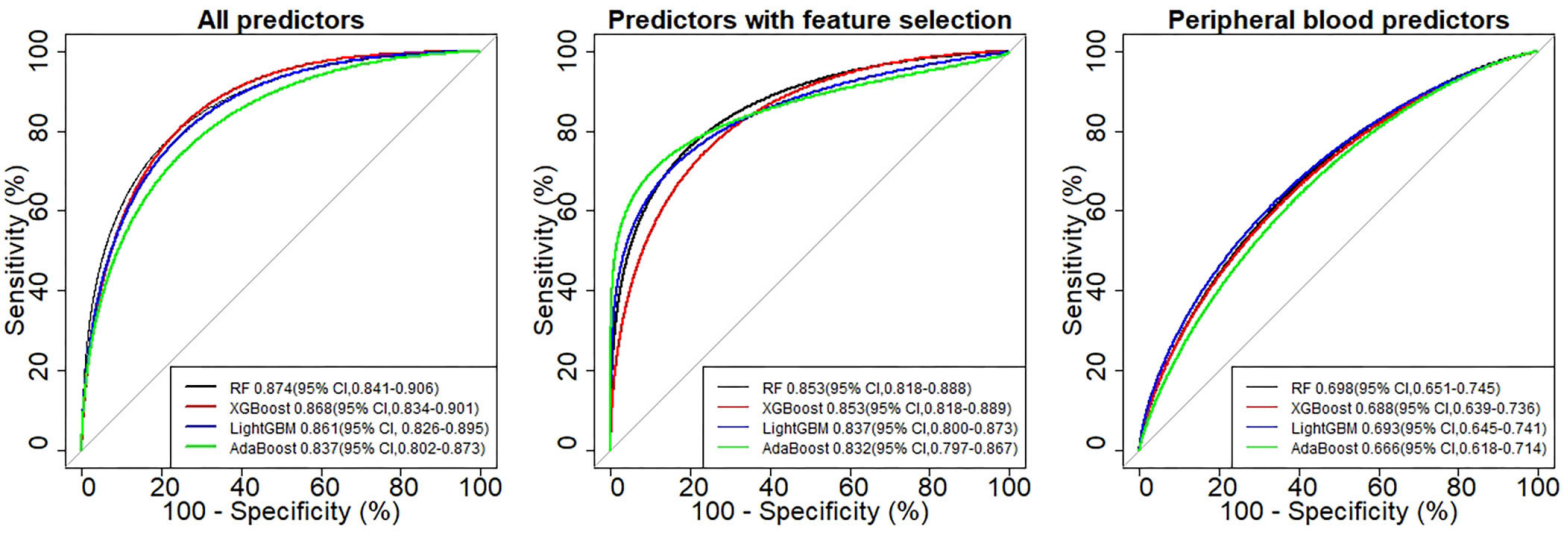
ROC curve of four models in different categories.

In Table 4, the metrics of three models were compared in terms of SEN, SPE, AUC, etc., in the test cohort. The SEN and precision are indicators to measure the positive predictive performance of the model. In the first and second models, the SEN indicator exceeds 0.7, and the precision indicator reaches 0.9, suggesting that the model we established can well identify malignant patients from thyroid tumor patients. The SPE is an indicator of the model's negative predictive performance, and in our study, the highest SPE was 0.892, indicating that our model could also predict patients with benign thyroid tumor well. The AUC is a comprehensive indicator for comparing prediction performance. Among the three models constructed with different predictors, the first model including all predictors performed best with the highest AUC of 0.874 (95% CI, 0.841, 0.906). The second model had the highest AUC of 0.853 (95% CI, 0.818, 0.889; Figure 3). However, we performed the Delong test on the optimal AUC of the first and second models (*z* = 1.65, *P* = 0.099), and the results showed that the difference was not statistically significant. The third model selects peripheral blood predictors, and the best AUC is 0.698 (95% confidence interval, 0.651, 0.745). In the third model, we selected biomarkers in patients' peripheral blood to establish a prediction model, and the performance of the model is inferior to the first and second models. Biomarkers in peripheral blood are easy to obtain, and the AUC of the model is close to 0.7, suggesting that it also has a certain predictive value.

**Table 4:** Performance evaluation table of three models.

**Categories**	**Algorithm**	**SEN**	**SPE**	**Precision**	**Recall**	**F1**	**AUC (95%CI)**
The first model	RF	0.790	0.886	0.949	0.790	0.862	0.874 (0.841–0.906)
	XGBoost	0.790	0.873	0.944	0.790	0.860	0.868 (0.834–0.901)
	LightGBM	0.734	0.892	0.948	0.734	0.827	0.861 (0.826–0.895)
	AdaBoost	0.723	0.855	0.931	0.720	0.812	0.837 (0.802–0.873)
The second model	RF	0.781	0.873	0.943	0.781	0.854	0.853 (0.818–0.888)
	XGBoost	0.754	0.873	0.941	0.754	0.837	0.853 (0.818–0.889)
	LightGBM	0.765	0.873	0.942	0.765	0.844	0.837 (0.800–0.873)
	AdaBoost	0.779	0.880	0.946	0.779	0.854	0.832 (0.797–0.867)
The third model	RF	0.671	0.645	0.836	0.671	0.744	0.698 (0.651–0.745)
	XGBoost	0.781	0.548	0.823	0.781	0.801	0.688 (0.639–0.736)
	LightGBM	0.624	0.705	0.849	0.626	0.721	0.693 (0.645–0.741)
	AdaBoost	0.626	0.651	0.828	0.626	0.713	0.666 (0.618–0.714)

**Figure 4:**
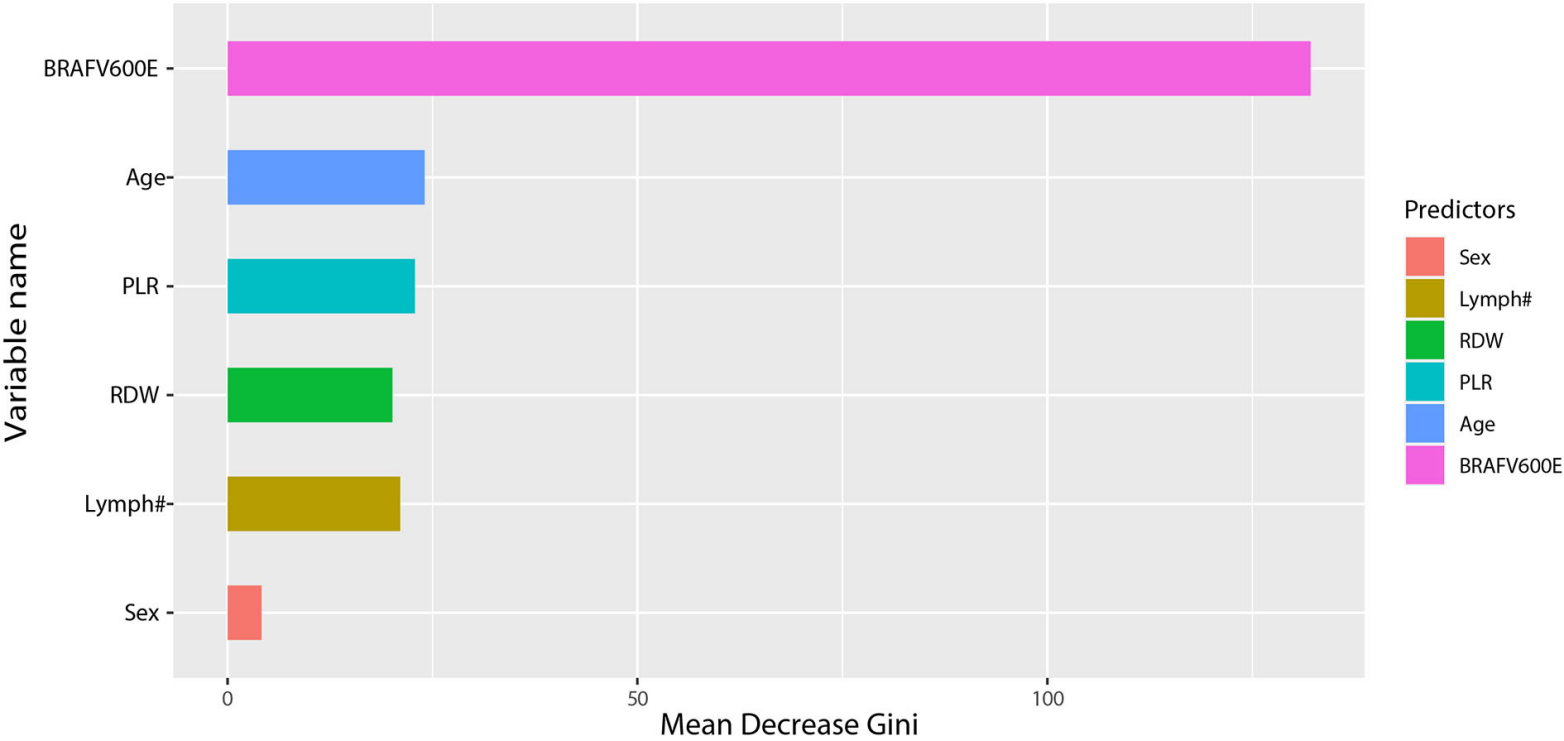
Importance ranking of prediction indicators after feature selection.

To balance the diagnostic performance and simplicity of the model, according to the comprehensive evaluation of the performance indicators of the model and the Delong test analysis, the second model, using the RF algorithm, was the best at predicting benign and malignant thyroid tumors. The importance ranking of predictors in the RF algorithm is as follows: BRAFV600E, age, PLR, RDW, Lymph#, and sex (Figure 4).

## Discussion

In this study, we developed the ML-based predictive models to identify benign and malignant thyroid nodules. The current gold diagnostic standard for thyroid tumors meeting appropriate criteria is a cyto-pathologic assessment of FNAB. However, high operator requirements were needed in FNAB, and the accuracy of diagnosis largely depends on the operator's personal level of experience. Therefore, it is crucial to provide more objective and direct parameters that can help with the identification of benign and malignant thyroid lesions. Thus, predictors including *BRAFV600E* gene mutation, Lymph#, Neu#, RDW, PLT, NLR, PLR, ALP, PTH, and clinical characters of patients were enrolled and the ML algorithm was used to predict benign and malignant thyroid tumors in our study.

Recent advances in understanding the molecular pathogenesis of thyroid tumors have enabled the application of molecular tests to provide more objective information and play a role in making more personalized clinical treatments (16). A large number of biomarkers such as BRAFV600E, RAS, EIF1AX, PIK3CA, PTEN and AKT1, SWI/SNF, ALK, and CDKN2A, have been excavated, demonstrating the potential of molecular diagnostic detection(17). Nevertheless, the BRAFV600E is the most prevalent mutation detected in PTC, with an average frequency of 60%−70%, and the tests for BRAFV600E mutation are commonly available in the current clinical practice (18). The BRAFV600E protein kinase has received extensive attention because of its function in promoting cell proliferation, growth, and division, and numerous studies have investigated the relationship between the BRAFV600E mutations and various clinicopathological features. *In vitro* tests have shown a high concordance between the BRAFV600E mutations and the aggressive characteristics of PTC, while clinical trials have shown contrasting results, making it controversial whether the BRAFV600E mutations can be used as an aggressive marker for PTC. Most studies suggest that the BRAFV600E mutations are associated with worse clinical pathology, such as lymph node metastasis, distant metastasis, worse tumor stage, aggressive subtype, tumor size, male, and old age, and therefore, recommend the central lymph node dissection based on total thyroidectomy with more stringent radioiodine therapy and a close follow-up after surgery (19). However, some studies did not find such an association (20). The differences in these studies may be due to the different sample sizes included in the studies, epidemiological characteristics of the patients, papillary carcinoma subtypes, types of specimens used for molecular testing, and testing methods. In this study, the *BRAFV600E* gene mutation status was important for all algorithms, which is consistent with a recent study. The BRAFV600E mutation has both high specificity and sensitivity to predict thyroid malignancy in the Chinese population. It can accurately complete cytopathology in the guidance of thyroid surgery (21). In our study, the diagnostic performance accuracy of the *BRAFV600E* gene was 0.810, and the AUC was 0.827, which had a high-diagnostic value.

The peripheral blood routine test and the blood biochemical test have major advantages over the traditional pathological test of tumor lesions in terms of quick and simple sample acquisition, low collection cost, minimal trauma, and preoperative detection, which should be paid more attention to in research (22). Lymph#, Neu#, RDW-CV, PLT, NLR, PLR, ALP, PTH, and other related indicators can quickly and accurately detect the values of blood, in order to effectively indicate abnormalities of infection, anemia, and cruor. In recent years, a wide variety of blood indicators with different changes were concerned and discussed in the study of malignant tumor diseases. The preoperative NLR and RDW–CV are convenient, practical, and easily measured biomarkers for clinical diagnosis and prognostic assessment of patients with esophageal cancer. Moreover, the NLR was more effective than RDW–CV, acting as an independent prognostic biomarker for esophageal cancer (23). On the contrary, the RDW–CV has attracted more attention in cervical, ovarian, and endometrial cancer as studies have shown the hierarchical independent relationship between the RDW and these kinds of cancers (24). The preoperative blood count from peripheral blood may provide prognostic value in patients with pathologic stage I NSCLC undergoing surgical resection. Of significance in patients with pT1 N0 NSCLC, the high lymphocyte count and high platelet count were associated with higher recurrence (25). Even the NLR, PLR, and LMR, which are the derived indexes of peripheral whole blood cell counts, were developed into new indexes, and have fairly good values of prognostic(26–28). However, the values of NLR and PLR to distinguish between benign and malignant of thyroid nodules is still controversial. Our study found that the Lymph#, RDW–CV, and PLR were statistically different between benign and malignant thyroid nodules (*P* < 0.05).

Recently, the ML algorithms have been extensively used in the medical field, emerging as a powerful tool in dealing with many health care problems. In our study, the ML-based model for diagnosing benign and malignant thyroid tumors showed the highest AUC of 0.874 (95% CI, 0.841, 0.906), which suggests that our model has a high value in diagnosing benign and malignant thyroid tumors. To evaluate the accuracy and simplicity of the model, feature selection is often used to screen indicators with predictive value. We screened out six predictors from 12 predictors by the univariate analysis method. Compared with the inclusion of 12 predictors, the model established by these six predictors also has good predictive performance and was identified as the optimal model. From the perspective of algorithm selection, when the indicators contained in the model are consistent, the performance of the four algorithms is not significantly different. One of the reasons is that if there is a clear correlation between the independent and dependent variables, then most ML algorithms can handle this nonlinear relationship and have good predictive performance. At present, many scholars have studied the use of artificial intelligence algorithms to accurately identify benign and malignant thyroid tumors (Table 5). The performance of our model is inferior to that of Hong-Bo Zhao, Sui, Peng et al., and similar to that of Masuda, Kim, Su Yeon Ko et al. Current researches mainly use ultrasound or CT images combined with intelligent algorithms to accurately diagnose benign and malignant thyroid tumors, and has excellent performance. In general, CT and ultrasound images have better predictive performance because they contain more information about benign and malignant tumors. However, from the perspective of patient's genetic markers and peripheral blood markers, our predictors are easy to obtain and has good value in identifying benign and malignant thyroid tumors.

**Table 5:** Comparison of the newly created model with the existing model.

**Title**	**Authors**	**Algorithms**	**Parameters**	**AUC**
Machine Learning for Identifying Benign and Malignant of Thyroid Tumors: A Retrospective Study of 2,423 Patients (final model)	Yuan-yuan Guo.et al	Machine learning (Random forest)	Sex, age, Lymph#, PLR, RDW, BRAFV600E	0.853 (95% CI, 0.818,0.888)
Deep learning-based artificial intelligence model to assist thyroid nodule diagnosis and management: a multicentre diagnostic study(13)	Sui, Peng. et al	Deep learning (ResNet, ResNeXt, DenseNet)	Ultrasound images	0.875 (95% CI, 0.871–0.880)
Machine learning to identify lymph node metastasis from thyroid cancer in patients undergoing contrast-enhanced CT studies (29)	Masuda et al	machine learning (Support Vector Machines)	CT images	0.86
Deep convolutional neural network for classification of thyroid nodules on ultrasound: Comparison of the diagnostic performance with that of radiologists (30)	Yeonjae et al.	Deep learning	Images of underwent US-guided fine-needle aspiration biopsy	0.83–0.86
Deep convolutional neural network for the diagnosis of thyroid nodules on ultrasound (31)	Yeon et al.	Deep learning (Convolutional Neural Network)	Ultrasound image	0.845, 0.835, and 0.850
A comparison between deep learning convolutional neural networks and radiologists in the differentiation of benign and malignant thyroid nodules on CT images (32)	Hong-Bo Zhao et al.	Deep learning (Convolutional Neural Network)	CT images	0.901–0.947

In conclusion, the prediction model established in this study can distinguish benign with the risk of identifying malignant thyroid nodules, which could be further developed into a clinical decision support system. Our study also had some limitations. First, all of the data come from southwest China, so there may be a selection bias. Second, only four algorithms were selected to establish the prediction model, therefore it is still necessary to try whether there are other better predictive algorithms. Third, the missing rate ≥30% of the variables were not included in the study. Therefore, further analysis is required to identify these factors related to identifying benign and malignant of thyroid nodules.

## Data availability statement

The original contributions presented in the study are included in the article/Supplementary material, further inquiries can be directed to the corresponding author.

## Author contributions

Y-yG, Z-jL, and PL took part in the research design and helped to draft the manuscript. J-xZ and CS contributed the acquisition of data. CD and JG performed the statistical analysis. All authors contributed to the article and approved the final manuscript.

## Funding

This work was supported by a grant for the Science and Technology and Health Commission program of Chongqing (2020FYYX157).

## Conflict of interest

The authors declare that the research was conducted in the absence of any commercial or financial relationships that could be construed as a potential conflict of interest.

## Publisher's note

All claims expressed in this article are solely those of the authors and do not necessarily represent those of their affiliated organizations, or those of the publisher, the editors and the reviewers. Any product that may be evaluated in this article, or claim that may be made by its manufacturer, is not guaranteed or endorsed by the publisher.
