# Supplementary material for: Machine learning for identifying benign and malignant of thyroid tumors: A retrospective study of 2,423 patients
Source: Front Public Health. 2022 Sep 14;10:960740. doi: 10.3389/fpubh.2022.960740 (PMC9515945; doi:10.3389/fpubh.2022.960740)
Supplement: Supplementary file 1 [file Data_Sheet_1.docx]

**Supplementary material**

Table1 Hardware device information in the development environment

| **Hardware Device** | **Device Information** |
| --- | --- |
| CPU | Intel(R) Core(TM) i7-10700K CPU @ 3.80GHz 3.79 GHz |
| RAM | Kingston DDR4 3200MHz 16GB |
| GPU | Intel(R) UHD Graphics 630 |
| SSD | WDS100T3X0C-00SJG0 |
| Mainboard | Gigabyte Z490 AORUS ELITE AC |

Table 2 Details of the three models

| **Model** | **Algorithms** | **Predictors** | **Memo** |
| --- | --- | --- | --- |
| The first model | RF, XGBoost, LightGBM, AdaBoost | Sex, BRAFV600E, Age, Lymph#, Neu#, NLR, PLR, RDW, PLT, RDW-CV, ALP, PTH | All predictors without feature selection |
| The second model | RF, XGBoost, LightGBM, AdaBoost | Sex, Age, Lymph#, PLR, RDW, BRAFV600E | With feature selection |
| The third model | RF, XGBoost, LightGBM, AdaBoost | Lymph#, Neu#, NLR, PLR, RDW, PLT, RDW-CV, ALP, PTH | Peripheral blood predictors |


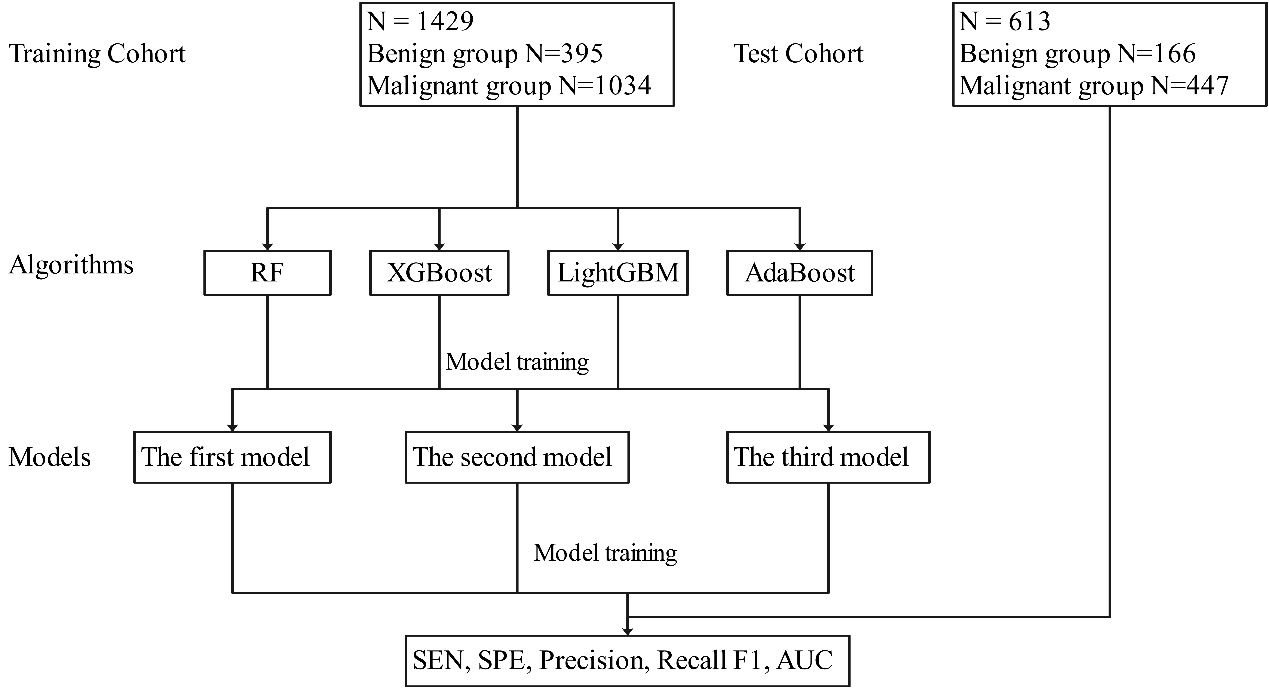


Fig 1. Model building flow chart

Table 3 Thyroid tumor malignancy group description

| Label | level | Malignant group |
| --- | --- | --- |
| Sex (%) | Female | 1124 (75.9) |
|  | Male | 357 (24.1) |
| Age(years) |  | 39.00 [32.00, 50.00] |
| Papillary carcinoma of thyroid gland (%) | no | 39 (2.6) |
|  | yes | 1442 (97.4) |
| Position (%) | Bilateral | 244 (16.5) |
|  | Isthmus | 9 (0.6) |
|  | left lobe | 580 (39.2) |
|  | Reft lobe | 644 (43.5) |
|  | Unknown | 4 (0.3) |
| Primary focus (%) | T1 | 506 (34.2) |
|  | T2 | 105 (7.1) |
|  | T3 | 472 (31.9) |
|  | Unknown | 398 (26.9) |
| Local lymph node metastasis(%) | N0 | 770 (52.0) |
|  | N1 | 395 (26.7) |
|  | N2 | 1 (0.1) |
|  | Unknown | 315 (21.3) |
| Distant metastasis (%) | M0 | 889 (60.0) |
|  | M1 | 2 (0.1) |
|  | Unknown | 590 (39.8) |
| Tumor diameter(mm) |  | 1. [5.00, 10.00] |
